# Endolymphatic hydrops in patients with migraine: a correlative study

**DOI:** 10.3389/fneur.2026.1882444

**Published:** 2026-06-26

**Authors:** Anne R. J. Péporté, Fabian Schön, Gustav Andreisek, Jatta Berberat, Franca Wagner

**Affiliations:** 1 Department of Radiology, Cantonal Hospital Frauenfeld, Frauenfeld, Switzerland; 2 Department of Radiology, Cantonal Hospital Münsterlingen, Münsterlingen, Switzerland; 3 Institute of Diagnostic and Interventional Radiology, University of Zurich, Zurich, Switzerland; 4 Department of Diagnostic and Interventional Neuroradiology, Cantonal Hospital Aarau, Aarau, Switzerland; 5 Department of Otorhinolaryngology and Head and Neck Surgery, Inselspital, Bern University Hospital, Faculty of Medicine, University of Bern, Bern, Switzerland

**Keywords:** endolymphatic hydrops (EH), infratentorial microbleeds, Ménière’s disease (MD), migraine imaging biomarkers, MRI, perivascular spaces, vestibulocochlear disorders, white matter lesions (WML)

## Abstract

**Objective:**

Endolymphatic hydrops (EH), Menière’s disease (MD) pathological hallmark, is visualized via 3 T delayed phase MRI. This study examines MRI correlations between EH severity and migraine brain biomarkers.

**Methods:**

Retrospective review of 144 inner ear MRI scans yielded 108 patients with confirmed EH. Two blinded radiologists graded EH, assessed migraine markers (white matter lesions (WML), frontal predominance of WML, enlarged perivascular spaces), and measured cranial nerve volumes. Binary logistic regression tested associations.

**Results:**

Mean age 53.6 ± 15.8 years (56.5% female). Grade 2 vestibular EH predominated bilaterally; grade 2 cochlear EH affected 34–43%. No EH associations with migraine biomarkers or cranial nerve volumes were found (all *p* > 0.05). Left vestibular EH grade 1 linked to sudden hearing loss (OR 5.205, *p* = 0.018); left cochlear EH grade 1 to aural fullness (OR 5.019, *p* = 0.016).

**Conclusion:**

EH severity was not associated with established migraine MRI biomarkers in this cohort. These findings support the concept that EH and migraine-related structural brain changes may represent distinct imaging phenomena.

## 1 Introduction

Endolymphatic hydrops (EH) involves pathological distension of the membranous labyrinth from excess endolymph, the potassium-rich fluid within it. Patients typically experience recurrent vertigo, fluctuating sensorineural hearing loss (SNHL), tinnitus, and aural fullness (1, 2). High-resolution 3 T MRI with delayed post-gadolinium sequences now allows *in vivo* visualization and grading of EH, including vestibular-cochlear ratios, herniation, and perilymphatic enhancement patterns key to Menière’s disease (MD) (3–6). Previously based solely on clinical and audiometric criteria, EH diagnosis has evolved over the past decade, incorporating MRI findings into the latest *Japan Society for Equilibrium Research guidelines on Menière’s disease* (7).

Migraine, a prevalent neurological disorder affecting approximately 15% of the population with significant global disability burden (8, 9), manifests as episodic (episodic migraine; <15 headache days/month) or chronic migraine (chronic migraine; ≥15 days/month with ≥8 meeting migraine criteria), often ± aura (visual, sensory, speech disturbances, or vertigo) (10–12). Vestibular migraine (VM) shares striking symptom overlap with MD—recurrent vertigo, unilateral hearing loss, tinnitus, aural fullness—prompting frequent misdiagnosis. According to a recent study by Kirsch et al. (13), 32% of VM patients show mild, bilateral vestibular-predominant EH versus MD’s severe unilateral cochlear-vestibular pattern, raising the question of whether EH severity correlates with migraine brain biomarkers. Potential shared mechanisms include Calcitonin gene-related peptide-mediated neurogenic inflammation affecting both endolymph homeostasis and central small vessel reactivity, or trigeminovascular activation linking peripheral hydrops to cortical spreading depression (8, 14).

Structural brain MRI reveals migraine-specific findings assessable via standard sequences: white matter lesions (WML) (Fazekas ≥1), frontal WML predominance and enlarged perivascular spaces—reflecting small vessel disease, iron deposition and glymphatic impairment (15–19).

This study investigates MRI correlations between EH and migraine biomarkers in an EH-confirmed cohort. Clarifying whether EH severity co-varies with established migraine MRI markers is a first step toward understanding potential shared mechanisms: identifying EH-migraine biomarker co-occurrence could resolve the MD-VM misdiagnosis rate (20) and guide precision therapy—avoiding ineffective VM treatments when severe unilateral EH confirms MD.

## 2 Methods

### 2.1 Patient population

The study was approved by the Ethics Committee of Eastern Switzerland (BASEC-ID: Req-2024-01599 EKOS 24/236). Individual informed consent was waived due to the retrospective nature of the study consistent with the local ethical guidelines. A retrospective review of our institutional digital database was carried out for delayed contrast-enhanced MRI scans of the inner ear for hydrops imaging between January 2020 and June 2025.

Exclusion criteria included temporal bone pathology (local/systemic diseases directly involving the temporal bone, *n* = 4), severe motion artifacts (*n* = 11), poor image quality due to severe distortion (*n* = 7), and incomplete MR imaging datasets (*n* = 2). Inclusion criteria required age >18 years and complete high-resolution 3 T MRI protocols including both pre- and 4-h post-contrast sequences covering the internal auditory canal (IAC) and inner ear structures.

From 144 scans in 132 patients, 36 were excluded, yielding a final cohort of 108 patients with MRI-confirmed EH, as previously characterized (21, 22). This represents the largest single-center EH cohort with comprehensive brain imaging assessment to date.

### 2.2 MRI acquisition

All participants underwent 3 Tesla MRI scanning (Siemens Magnetom Vida 3 Tesla, Philips Achieva 3 Tesla) using standardized protocols optimized to visualize inner ear structures, the vestibulocochlear nerve complex, and the endo- and perilymphatic spaces. Acquisitions included 3D high-resolution T2-weighted sequences and delayed post-gadolinium sequences (3D FLAIR and 3D T2w inversion recovery sequence 4 h post-contrast) (Table 1).

**Table 1:** MRI scanning parameters.

Sequence	Plane	Field of view (mm)	Echo time (ms)	Repetition time (ms)	Flip angle (°)	Slice thickness (mm)
T2	3D tra	150 × 150	Shortest	1,500	90	0.8
FLAIR 4 h post-contrast	3D tra	250 × 250	340	4,800	40	1.0
T2 inversion recovery 4 h post-contrast	3D tra	75 × 150	177	6,000	180	0.8

Example of detailed scanning parameters of the specific sequences of our inner ear endolymphatic hydrops MRI protocol at a 3 Tesla MRI Scanner (Philipps Magnetom Achieva).

Tra, transverse; cor, coronal; mm, millimeters; ms, milliseconds.

### 2.3 MRI assessment and volumetry

#### 2.3.1 MRI migraine biomarkers assessment

Brain MR images from EH patients were systematically assessed for migraine markers by two raters—AP (a board-certified neuroradiologist holding the European Diploma in Head and Neck Radiology, with more than 6 years’ experience in head and neck imaging) and FS (a general radiologist with more than 10 years’ experience in general radiology) using validated visual rating methods on standard sequences: coronal/sagittal T1w, axial/sagittal FLAIR/T2w (WML), and T2w (perivascular spaces). Disagreements between both readers were resolved by a third senior reader (FW, board-certified neuroradiologist and European Diploma in Head and Neck Radiology holder with more than 20 years’ experience in neuroradiology, head and neck, and inner ear imaging).

Image-based migraine markers included WML (Fazekas scale), frontal predominance of WML and enlarged perivascular spaces (see Table 2).

**Table 2:** Migraine markers assessed.

Marker	MRI sequence	Assessment method
WML	Axial/sagittal FLAIR/T2w	Fazekas visual scale
Frontal predominance of WML	Axial/sagittal FLAIR/T2w	Distribution pattern
Enlarged perivascular spaces	T2w	Visual grading

Example of detailed scanning parameters of the specific sequences of our inner ear endolymphatic hydrops MRI protocol at a 3 Tesla MRI Scanner (Philipps Magnetom Achieva).

FLAIR, fluid-attenuated inversion recovery; T2w, T2-weighted; WML, white matter lesions.

**Figure 1:**
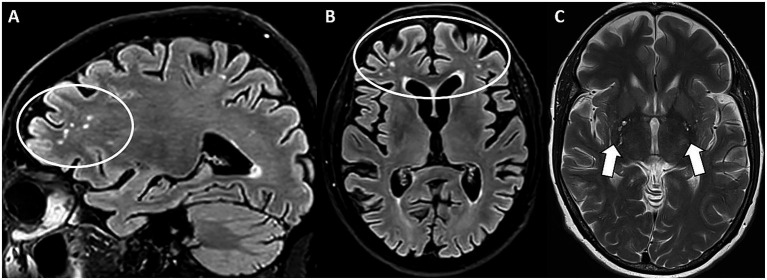
Example of MRI migraine markers. Sagittal **(A)** and axial **(B)** FLAIR and axial T2w **(C)** MR images of a patient with known migraine. There are confluent white matter hyperintensities [Fazekas grade 2, white circles in **(A)** and **(B)**] predominantly in frontal subcortical and deep white matter regions. Symmetrically enlarged perivascular spaces are seen in the posterior limb of the internal capsule [arrows in **(C)**].

An example of migraine markers MR assessment is given in Figure 1.

#### 2.3.2 Cranial nerve volumetric measurement

Cochlear and vestibular nerve volumes were quantified by AP using semi-automated segmentation software package (Syngo.via, Siemens Healthineers, version VB80D) for volumetric measurements. Bilateral cochlear nerve and vestibular nerve complex volumes were obtained by axis-corrected measurement from the cerebellopontine angle to the IAC fundus. Volume of interest regions were inserted along the entire course of the cranial nerves. The cumulative area of each MRI slice was then calculated by the software to obtain the entire nerve volume. Edge blurring (penumbra effect) was addressed by contouring at the midpoint between central low-signal nerve core and surrounding high-signal CSF.

The volume of the common vestibular nerve was quantified without measuring the superior and inferior vestibular nerve volumes separately. These two branches often remain fused throughout their course through the IAC. Anatomical studies have shown that the vestibular nerve divides into its superior and inferior branches only near the lateral end of the canal, typically at or just beyond the falciform crest. For most parts of their intrameatal course, these divisions cannot be distinctly visualized or separated, either by cadaveric dissection or by imaging, because they are commonly fused. In many specimens, the superior and inferior vestibular nerves appear as a single structure (the common vestibular nerve) and only turn clearly distinguishable at the fundus of the IAC. Because of this anatomical continuity, volumetric measurements of the common vestibular trunk are most reliable, as attempting to differentiate and separately measure the individual branches would not be consistently feasible or reproducible and do not accurately reflect the true anatomy of the nerves (23, 24).

**Figure 2:**
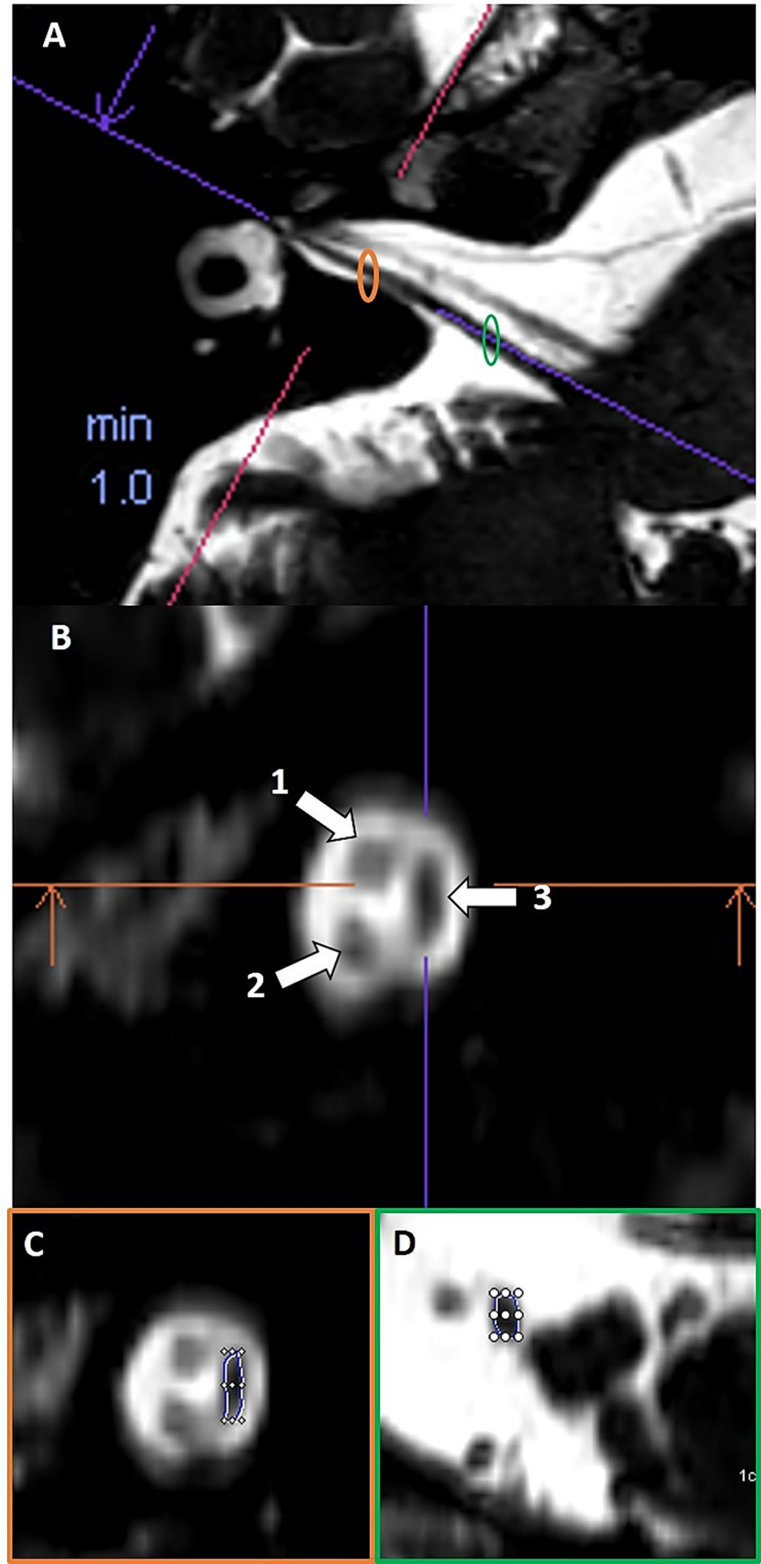
Example of volumetric measurement of the common vestibular nerve. Axial T2w SPACE image **(A)** at the level of the internal auditory canal showing the orientation of the common vestibular nerve and location of the oblique sagittal 0.8 mm multiplanar reformat from which neural volumetric measurements were obtained. The corresponding oblique sagittal reformatted image **(B)** at the fundus of the internal auditory canal is positioned just lateral to the cochlear aperture. The facial nerve (1), cochlear nerve (2) and the common vestibular nerve (3) are marked. Panels **(C)** and **(D)** show sections of the volumetric drawing of the common vestibular nerve, one example in the intrameatal segment (orange ellipse) and one example in the cisternal segment (green ellipse).

An example of cranial nerve volumetric measurement is given in Figure 2.

#### 2.3.3 EH grading

Two readers (AP and FS) independently graded cochlear and vestibular EH, according to established MRI criteria (25). Ears were categorized into one of four distinct grades (Grade 0/no EH, Grade 1, Grade 2, and Grade 3 vestibular EH; Grade 0/no EH, Grade 1 and Grade 2 cochlear EH), as outlined by Bernaerts et al. (25). Disagreements between both readers were solved by a third senior reader (FW). For grading vestibular EH, the saccule and utricle were analyzed at the most inferior level of the vestibule to avoid artifactual fusion or apparent enlargement of these structures that can occur when evaluating more superior slices. All readers were blinded to clinical data. Control ears were defined as ears graded 0 (no EH) according to Bernaerts et al.’s established semiquantitative MRI grading system for EH (25). This imaging-based definition does not exclude the presence of subtle or sub-visual volumetric EH, which was not quantified in this study.

**Figure 3:**
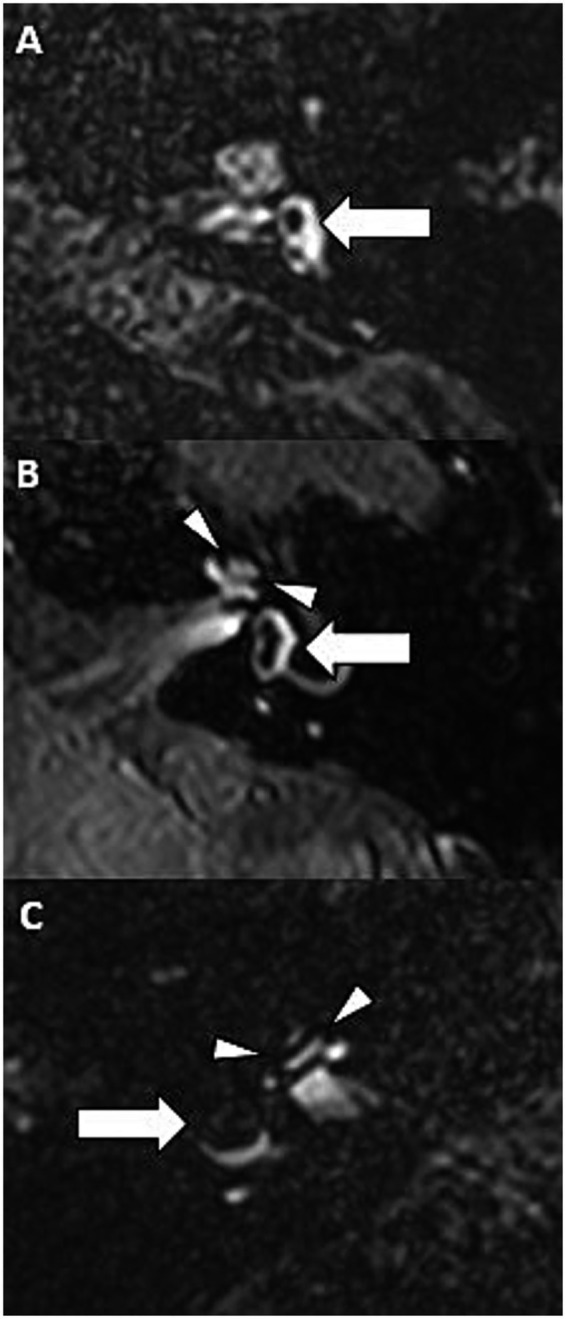
Examples of EH grading. Delayed post-gadolinium axial 3D T2 inversion recovery **(A,C)** and delayed post-gadolinium axial 3D FLAIR images. **(A)** There is enlargement of the saccule on the left (white arrow) without fusion with the utricle, consistent with a vestibular endolymphatic hydrops grade 1. **(B)** Asymmetrical perilymphatic enhancement, more pronounced on the left. Nodular hypo-intensities in the periphery of the apical and midturn of the cochlea on the left (arrow heads). No bandlike hypo-intensities in the basal turn of the cochlea making this a cochlear hydrops grade 1. The left saccule is enlarged and is just confluent with the utricule with a remaining peripheral rim enhancement, making this a vestibular hydrops grade 2. **(C)** There is fusion of the utricle and saccule on the right and the perilymphatic enhancement is no longer visible (arrow), compatible with a vestibular hydrops grade 3. The scala vestibuli is fully obliterated due to the distended cochlear duct (arrow heads), cochlear hydrops grade 2.

An example of EH grading is given in Figure 3.

### 2.4 Clinical data and symptom assessment

Data on clinical symptoms extracted from patient records included presence or absence of hearing loss, sudden hearing loss, right/left-sided hearing loss, bilateral hearing loss, SNHL, vertigo, tinnitus, and aural fullness.

Clinical referrals for EH MRI covered a spectrum of vestibulocochlear presentations, including suspected MD or hydropic ear disease, recurrent or fluctuating vertigo, sudden or progressive SNHL, tinnitus, and aural fullness. Because diagnostic labels (e.g., “MD,” “VM,” “overlap syndrome”) were assigned in routine care by different clinicians and formal Bárány or AAO-HNS/Japan Society criteria were not systematically documented, we did not stratify patients into MD, VM, SNHL, or other diagnostic categories. This diagnosis-agnostic design was chosen *a priori* to avoid misclassification bias from heterogeneous chart diagnoses and to enable an imaging-based analysis of the relationship between EH severity and migraine MRI biomarkers in a real-world hydropic ear cohort. The majority of referrals were for suspected MD or hydropic ear disease.

### 2.5 Statistical analysis

Statistical analysis was conducted using SPSS (IBM Corp., Armonk, NY, USA) V 29.0.1.0. The effects of image-based migraine markers and clinical symptoms to EH grading were tested using binary logistic regression. Adjusted odds ratios with 95% confidence intervals are reported for included predictors.

Image-based migraine markers included: Fazekas Scale (0 = absent WML, 1 = punctate foci of WML, 2 = beginning confluence of WML, 3 = large confluent areas of WML), frontal predominance of WML, and enlarged perivascular spaces.

Clinical symptoms included: hearing loss, sudden hearing loss, right/left-sided hearing loss, bilateral hearing loss, SNHL, vertigo, tinnitus and aural fullness.

## 3 Results

### 3.1 Patient population

A study cohort of 108 patients diagnosed with EH met the inclusion criteria. Mean age was 53.6 ± 15.8 years and there was a slight female predominance (61/108; 56.5%).

### 3.2 MRI assessment and volumetry

#### 3.2.1 EH grading

In the vestibular system, grade 2 EH was observed in 71.3% (77/108) of right and 60.2% (65/108) of left vestibules, while grade 1 EH was seen in 15.7% (17/108) on the right and 21.3% (23/108) on the left. Only one participant (0.9%) demonstrated grade 3 EH on the left side, and no cases of grade 3 EH were identified on the right.

Similarly, in the cochlea, grade 2 EH predominated, affecting 42.6% (46/108) on the left and 34.3% (37/108) on the right. Grade 1 EH was present in 19.4% (21/108) of right cochleae and 18.5% (20/108) of left cochleae, while grade 3 EH was rare, observed in only one right-sided case (0.9%) and none on the left.

No correlation between the patient age and EH grading was found.

#### 3.2.2 EH grading vs. cranial nerve volume, image-based migraine markers and clinical markers

Binary logistic regressions revealed no significant associations between migraine MRI markers (WML, frontal WML predominance, enlarged perivascular spaces) and vestibular or cochlear EH grades (all *p* > 0.05). No correlations existed between EH grades and cranial nerve volumes (all *p* > 0.05).

Binary logistic regression showed significant odds ratio for association between left cochlear EH grade 1 and aural fullness (OR 5.019 [95%CI 1.343–18.748] *p* = 0.016). Left vestibular EH grade 1 was significantly associated with sudden hearing loss (OR 5.205 [95% CI 1.327–20.411] *p* = 0.018).

A summary of the results is presented in Table 3.

**Table 3:** Summary of demographic characteristics, quantitative imaging findings, and prevalence of radiological migraine markers in 108 patients with endolymphatic hydrops.

Category	Variable	Mean (SD)	*N* (%)
Demographics	Age (years)	53.6 (15.8)	
	Sex (female)		61 (56.5%)
Imaging: CN volumetry	Left cochlear nerve volume (mm^3^)	9.52 (4.92)	
	Right cochlear nerve volume (mm^3^)	9.10 (5.90)	
	Left vestibular nerve volume (mm^3^)	18.95 (9.7)	
	Right vestibular nerve volume (mm^3^)	19.78 (10.18)	
	Left facial nerve volume (mm^3^)	12.35 (5.45)	
	Right facial nerve volume (mm^3^)	13.71 (5.83)	
MRI: EH grading	Vestibular EH grade 1, left		23 (21.3%)
	Vestibular EH grade 1, right		17 (15.7%)
	Cochlear EH grade 1, left		20 (18.5%)
	Cochlear EH grade 1, right		21 (19.4%)
	Vestibular EH grade 2, left		65 (60.2%)
	Vestibular EH grade 2, right		31 (28.7%)
	Cochlear EH grade 2, left		46 (42.6%)
	Cochlear EH grade 2, right		37 (34.3%)
	Vestibular EH grade 3, left		1 (0.9%)
	Vestibular EH grade 3, right		0 (0%)
	Cochlear EH grade 3, left		0 (0.0%)
	Cochlear EH grade 3, right		1 (0.9%)
MRI: migraine markers	WML (Fazekas ≥1)		60 (55.6%)
	Frontal predominance of WML		18 (16.7%)
	Enlarged perivascular spaces		25 (23.1%)
Clinical symptoms	Vertigo		75 (69.4%)
	Sensorineural hearing loss		76 (70.4%)
	Tinnitus		48 (44.4%)
	Sudden hearing loss		23 (21.3%)

Data are presented as mean (standard deviation) for continuous variables and number (percentage) for categorical variables.

SD, standard deviation; CN, cranial nerve; EH, endolymphatic hydrops; WML, white matter lesions.

## 4 Discussion

This study represents the first comprehensive MRI-based analysis exploring correlations between EH severity and established migraine-related brain imaging biomarkers in a large single-center cohort of 108 patients. The absence of significant associations between EH grades and migraine MRI features—including total and frontal-predominant WML, enlarged perivascular spaces, or regional WML distribution—has important mechanistic implications. Rather than simply reflecting unrelated findings, this dissociation does not support a direct relationship between EH severity and established migraine MRI biomarkers, and argues against a common microvascular or trigeminovascular substrate between EH and migraine-associated central white matter injury.

Mechanistically, migraine-related brain alterations arise from chronic small vessel disease, blood–brain barrier dysfunction, glymphatic impairment, and iron deposition secondary to repeated cortical spreading depression episodes (8, 16–19). In contrast, EH represents a peripheral process marked by disturbed ionic transport, impaired endolymph resorption within the endolymphatic sac, aquaporin dysregulation, and a resulting imbalance of longitudinal endolymphatic pressure (1, 2). The lack of correlation suggests that these imaging manifestations may arise from different biological processes.

In our previously analyzed cohort, EH frequently co-occurred with MRI markers of idiopathic intracranial hypertension (IIH) (21), suggesting that IIH-related pressure dysregulation may contribute to headache in at least a subset of EH patients. However, EH is a downstream manifestation of several inner ear disorders, including vestibular migraine, and migraine – with its characteristic headache features and (vestibular) symptoms—remains an important and clinically distinguishable cause of headache in this population. Our data therefore support IIH as a potential additional contributor to headache burden in EH, but do not allow us to determine the relative contribution of IIH versus (vestibular) migraine in individual patients, underscoring the need for careful clinical differentiation of headache types.

Frontal-predominant WMLs are consistently interpreted as imaging markers of migraine-related small vessel injury. Their burden correlates with migraine attack frequency, aura presence, disease duration, and endothelial dysfunction (8, 15, 26–29). If EH were downstream of chronic trigeminovascular activation or small vessel pathology, a severity-dependent relationship between EH grade and frontal WML load would be expected. Our observation of no such gradient therefore carries conceptual weight, as it suggests that EH does not evolve as a consequence of cumulative migraine-related microvascular injury. This negative result is informative, because it delineates EH as a peripheral phenomenon, that is mechanistically independent of migraine-related cerebral changes.

An alternative hypothesis proposes that migraine-related vasospasm, inner ear ischemia or CGRP-mediated labyrinthine microvascular dysregulation might induce secondary EH (30–35). Our findings do not provide imaging evidence supporting this model. No pattern of EH severity mirrored migraine biomarker expression, indicating that chronic microangiopathic or glymphatic mechanisms linking migraine brain injury to secondary EH are unlikely. This reinforces the view that EH is not a vascular consequence of migraine but rather reflects primary labyrinthine dysfunction.

From a clinical perspective, this mechanistic dissociation is diagnostically valuable. EH severity did not parallel migraine biomarker burden, confirming EH’s behavior as a peripheral marker, while migraine imaging changes remained centrally determined. These findings are consistent with previous work suggesting that EH may provide useful information in the differential diagnosis between MD and VM. Our findings complement previous prospective work demonstrating near-perfect discrimination of MD and VM based on cochlear–vestibular EH patterns (36), extending that evidence by showing that EH severity is independent of migraine-related brain injury.

The occasional detection of mild EH in VM remains incompletely understood. Potential contributing mechanisms include transient perturbations of blood–labyrinth barrier permeability, autonomic dysregulation influencing endolymph homeostasis, or post-ictal vestibular dysfunction (13, 36–38). Yet, our data argue against any causal link between these peripheral changes and migraine-associated structural brain injury.

Clinical symptoms correlations strengthen EH’s peripheral specificity. Left vestibular EH grade 1 associated with sudden hearing loss (OR 5.205, *p* = 0.018) and left cochlear EH grade 1 with aural fullness (OR 5.019, *p* = 0.016) align with MD’s classic otologic triad, reinforcing EH as a peripheral biomarker rather than VM surrogate. No cranial nerve volume correlations further exclude central neurodegenerative contributions. Our cranial nerve volumetry in this cohort revealed no significant EH associations, consistent with our separate volumetric analysis focused on nerve morphometry, which also found that EH severity does not significantly affect cochlear or vestibular nerve volumes or symptom prevalence (22), and aligns with external reports that normalized cochlear nerve calibre does not differ significantly in EH (39). These convergent negative findings indicate that volumetric MRI of the vestibulocochlear nerve, while feasible and reproducible, is unlikely to provide an additional sensitive biomarker of EH beyond established hydrops imaging and clinical assessment.

An additional finding of our study was the absence of a significant correlation between patient age and EH grading. This result contrasts with previous studies that have suggested a higher prevalence or greater severity of EH in older patients with hydropic ear disease, possibly reflecting cumulative inner ear injury or long-standing endolymphatic dysfunction (40, 41). In our cohort, several factors may have attenuated an age–EH relationship, including the heterogeneous clinical indications for EH imaging and the relatively wide age distribution. Moreover, delayed gadolinium-enhanced MRI detects structural EH at a single time point and may not fully capture dynamic or duration-dependent changes in endolymph homeostasis. Thus, while our data argue against a strong, monotonic age effect on EH grade in this mixed cohort, they do not exclude more subtle age-related influences. Future prospective, age-stratified studies with well-defined disease duration and phenotype will be required to clarify whether specific hydropic ear subgroups exhibit age-dependent progression of EH.

Limitations include the heterogeneous clinical indications for EH imaging and the retrospective design, which precluded standardized migraine phenotyping (attack frequency, aura status) and formal diagnostic stratification into MD, VM, overlap syndromes, and isolated SNHL. Diagnostic labels in the medical records were assigned by different clinicians using non-uniform criteria and were therefore not used for subgroup analyses in order to avoid misclassification bias. As a consequence, our findings should be interpreted as imaging-based relationships between EH severity and structural migraine MRI biomarkers in a diagnosis-agnostic hydropic ear cohort, rather than as direct comparisons between rigorously phenotyped MD and VM groups.

We also lacked quantitative volumetry for WMLs and EH compartments and did not assess longitudinal progression or causal directionality. To refine these observations, future prospective multicentric studies with standardized clinical work-up and dedicated MD–VM cohorts should integrate quantitative EH and WML measurements and CSF pressure assessments.

Another limitation is the absence of a systematic extralabyrinthine assessment of the endolymphatic duct and sac (morphology, angle, and contrast enhancement). These features are increasingly explored for MD phenotyping and VM/MD differentiation, but they are not yet part of established EH criteria and are not integrated into the latest Japan Society for Equilibrium Research guidelines on MD. Future prospective studies with dedicated protocols should include endolymphatic duct and sac parameters to determine their incremental diagnostic value.

In conclusion, EH severity was not associated with established migraine-related MRI biomarkers, including frontal WML predominance and enlarged perivascular spaces, in this EH-confirmed cohort. Within the limits of our retrospective imaging design and lack of standardized VM/MD phenotyping, these findings do not support a clear association between EH severity and established migraine MRI biomarkers. MRI-based EH assessment therefore currently retains diagnostic specificity for MD rather than representing a radiological epiphenomenon of migraine-associated brain injury. Prospective further studies with standardized clinical phenotyping are required to clarify potential pathophysiological interactions.

## Data Availability

The raw data supporting the conclusions of this article will be made available by the authors, without undue reservation.
